# Metacommunity and phylogenetic structure determine wildlife and zoonotic infectious disease patterns in time and space

**DOI:** 10.1002/ece3.1404

**Published:** 2015-01-23

**Authors:** Gerardo Suzán, Gabriel E García-Peña, Ivan Castro-Arellano, Oscar Rico, André V Rubio, María J Tolsá, Benjamin Roche, Parviez R Hosseini, Annapaola Rizzoli, Kris A Murray, Carlos Zambrana-Torrelio, Marion Vittecoq, Xavier Bailly, A Alonso Aguirre, Peter Daszak, Anne-Helene Prieur-Richard, James N Mills, Jean-Francois Guégan

**Affiliations:** 1 Departamento de Etología, Fauna Silvestre y Animales de Laboratorio, Facultad de Medicina Veterinaria Zootecnia, Universidad Nacional Autónoma de MéxicoMéxico, Distrito Federal, México; 2 UMR MIVEGEC, Maladies Infectieuses et Vecteurs: Ecologie, Génétique, Evolution et Contrôle UMR 5290 CNRS-IRD-UM1-UM2, Centre de Recherche IRD34394, Montpellier Cedex 5, France; 3 Centre de Synthèse et d'Analyse sur la Biodiversité – CESAB13857, Aix-en-Provence Cedex 3, France; 4 Biology Department, Texas State UniversitySan Marcos, Texas; 5 EcoHealth AllianceNew York, New York; 6 Biodiversity and Molecular Ecology Department Research and Innovation Centre, Fondazione Edmund Mach, San Michele all'AdigeTrento, Italy; 7 INRA, UR346 Epidémiologie AnimaleSaint Genès Champanelle, France; 8 Department of Environmental Science and Policy, George Mason UniversityFairfax, Virginia; 9 Muséum National d'histoireNaturelle, DIVERSITASParis, France; 10 Population Biology, Ecology and Evolution Program, Emory UniversityAtlanta, Georgia

**Keywords:** Disease ecology, dispersal, evolution, metacommunity, One Health, phylogenetic structure, stochastic event

## Abstract

The potential for disease transmission at the interface of wildlife, domestic animals and humans has become a major concern for public health and conservation biology. Research in this subject is commonly conducted at local scales while the regional context is neglected. We argue that prevalence of infection at local and regional levels is influenced by three mechanisms occurring at the landscape level in a metacommunity context. First, (1) dispersal, colonization, and extinction of pathogens, reservoir or vector hosts, and nonreservoir hosts, may be due to stochastic and niche-based processes, thus determining distribution of all species, and then their potential interactions, across local communities (metacommunity structure). Second, (2) anthropogenic processes may drive environmental filtering of hosts, nonhosts, and pathogens. Finally, (3) phylogenetic diversity relative to reservoir or vector host(s), within and between local communities may facilitate pathogen persistence and circulation. Using a metacommunity approach, public heath scientists may better evaluate the factors that predispose certain times and places for the origin and emergence of infectious diseases. The multidisciplinary approach we describe fits within a comprehensive One Health and Ecohealth framework addressing zoonotic infectious disease outbreaks and their relationship to their hosts, other animals, humans, and the environment.

## Introduction

Anthropogenic environmental changes often erode biodiversity and disrupt ecosystem function (Dirzo et al. 2014). These changes can have numerous negative effects, including increased risk of emergence of new pathogens and alteration of transmission dynamics of endemic diseases (Foley et al. 2005). Given that the majority of infectious diseases affecting humans are of animal origin (Taylor et al. 2001), pathogens with zoonotic potential (micro- and macroparasites) transmissible between humans and animals including wildlife are of major concern. The link between animal and human diseases has sparked the introduction of new conceptual frameworks, such as “One Health” and “Ecohealth”, aimed at understanding the dynamics and drivers of diseases at the interface between humans, wildlife, domestic animals, and the environment. Within these frameworks, most studies on the ecology of zoonotic diseases caused by pathogens hosted by wildlife reservoirs and vectors (*henceforth* hosts) focus at the level of local populations and communities and the environment in which these interactions occur (Karesh et al. 2012). Very few consider dimensions at larger scales such as the landscape and regions that play an important role in infectious disease dynamics (Grenfell and Harwood 1997; McCallum and Dobson 2002). Hay et al. (2013) indeed noted that we have barely begun to understand human disease biogeography, as the spatial distribution of only seven of 355 known infectious diseases that affect humans, has been adequately characterized.

Wildlife hosts rarely function as discrete and isolated units. They establish ecological relationships with other species within a community, including pathogens, vectors, hosts, and nonhosts. Furthermore, each local community is embedded within a metacommunity, a series of local communities linked by dispersal (Leibold et al. 2004). The metacommunity concept was conceived to bridge local (site-specific) and biogeographic scales (Holyoak and Loreau 2006), but the application of this analytical framework is not limited to these scales (Mihaljevic 2012). Because a metacommunity encompasses assemblages of hosts and nonhosts that interact and influence pathogen spread and transmission, consideration of this level of organization is useful for investigating multihost pathogens for which population and metapopulation approaches are not sufficient. A clear example would be the Chagas disease in which trypanosomes have multiple mammalian reservoir species and triatomine vectors at any given landscape (Bern et al. 2011).

Landscape studies using a metacommunity framework can provide novel insights into the mechanisms of emergence of infectious diseases in wildlife including zoonoses. This is especially true in human-dominated landscapes because habitat loss and fragmentation have a direct effect on many ecological processes that may play a role in infectious disease emergence. These processes include dispersal (daily, seasonal, or annual movements), colonization (re-establishment of a population within a species' original range), invasion (establishing a population outside the species' original range), and local extinction of both pathogens and hosts (see Table1). In addition, pathogen evolution and adaptation to new hosts may be affected by anthropogenic pressures (Murray and Daszak 2013). Currently, although several studies highlight their importance (Mihaljevic 2012; Henriques-Silva et al. 2013; Maurer et al. 2013), little is known about how these processes drive the distribution and prevalence of infectious diseases at the landscape level. To contribute to understanding of landscape-level disease dynamics, we propose a conceptual model of three plausible mechanisms that may influence these dynamics.

**Table 1:** Definitions

Concept	Definition
Boundary clumping	One of the elements of metacommunity structure that describes how the edges of species boundaries are distributed along an environmental gradient
Coherence	One of the elements of metacommunity structure, it is the response of species to an environmental gradient quantified with the number of embedded absences of a species distribution among sites
Environmental filtering	Restriction of species that persist within a community on the basis of their tolerance of the abiotic environment
Host switching	The switching of parasitic organisms to novel hosts
Nestedness	Ranges of species that occupy a smaller portion of the environmental gradient are contained within the ranges of those that occupy a larger portion of the gradient
Niche based processes	Interspecific biotic interactions and abiotic conditions affecting persistence of species in a given community over time
Phylogenetic structure	Phylogenetic relatedness of co-occurring species in time and space
Species turnover	One of the elements of metacommunity structure that describes the number of species replacements along the metacommunity
Spillover transmission	Inter-species transmission from a maintenance host to a non-maintenance host
Spillback transmission	Transmission from a non-maintenance host back into the maintenance host species from which it originated

### Pathogen prevalence across landscapes

#### Dispersal, colonization, and extinction

**Figure 1:**
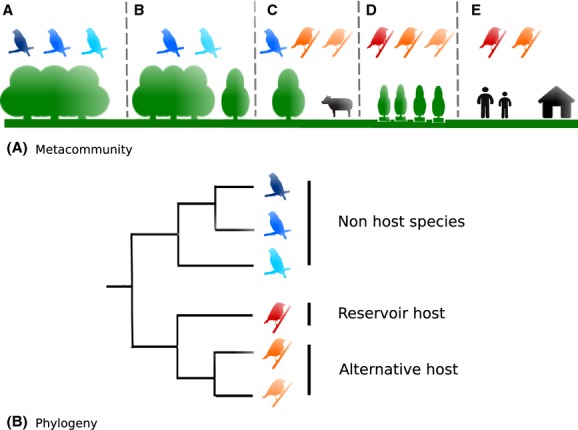
Conceptual model of ecological and evolutionary relationships within communities that regulate prevalence of infection with a zoonotic pathogen in the reservoir host. (A) A simplified metacommunity composed of five communities (A, B, C, D, E). Reservoirs (red) and related species (alternative host species in orange) maintain higher infection prevalence. The highest prevalence is expected in communities C, D, and E and the lowest in communities A and B with nonhost species in blue. (B) Phylogenetic tree of all species in the metacommunity.

Across the landscape, populations of a given species may colonize or go locally extinct due to the dispersal of individuals and local population dynamics (Hanski and Gilpin 1997). These processes may determine the genetic and phenotypic structure of the metapopulation of a species and several studies have demonstrated the importance of metapopulation structure on disease dynamics for persistence of pathogens in highly dynamic landscapes (Grenfell and Harwood 1997; McCallum and Dobson 2002). Likewise, dispersal of individuals and the subsequent colonization and extinction of populations of multiple species determine the structure of the communities within a metacommunity. However, much less is known about how metacommunity structure may determine disease dynamics across landscapes. Epidemiologic theory suggests that the proportion of individuals in a population that are infected by a pathogen (prevalence) is the outcome of transmission dynamics between infected and susceptible hosts (Anderson and May 1979). Likewise, at the landscape level, transmission of a pathogen between hosts of distant communities would be determined by dispersal, colonization, and local extinction of infected and susceptible hosts between these communities (Fig.1).

The relationship between metacommunity structure and infectious disease dynamics has not been examined from a theoretical perspective and analyses of empirical data are lacking. Using hosts as “habitat patches”, Mihaljevic (2012) called for integration of metacommunity theory to explore patterns in composition of microbiota communities. This insightful framework encompasses the ecology and evolution of the vast diversity of organisms living within hosts, including disease-causing micro- and macroparasites at a wide spectrum of nested levels of life organization from host organs to interhost subpopulations. Nevertheless, this framework neglects drivers of within-host assemblages at higher levels of spatial organization, such as distribution of host communities across landscapes. In theory, metacommunity structure is determined by stochastic processes, and the tolerance of species to ecological conditions (their niches) that occur in a set of communities linked by dispersal (Leibold et al. 2004; Presley et al. 2010). Conceptual models of species distributions over environmental or geographical gradients have a long history in ecology, with early research being mostly descriptive (Clements 1916; Gleason 1926; Whittaker 1965). More recently, a strict quantitative framework was developed and later refined to distinguish commonly observed patterns of species distributions among sites (Leibold et al. 2004; Ulrich et al. 2009; Presley et al. 2010). The core framework distinguishes between three idealized groups of metacommunity structures: random, nested, and antinested with several substructures possible within these last two categories (Table2). This framework provides an objective approach to determine which idealized structure best characterizes the host and nonhost species' distributions among sites. As theoretical foundations for each structure are idiosyncratic and distinct (Clements 1916; Gleason 1926; Diamond 1975; Tilman 1982), different hypotheses regarding the origin of metacommunity structure can be tested.

**Table 2:**
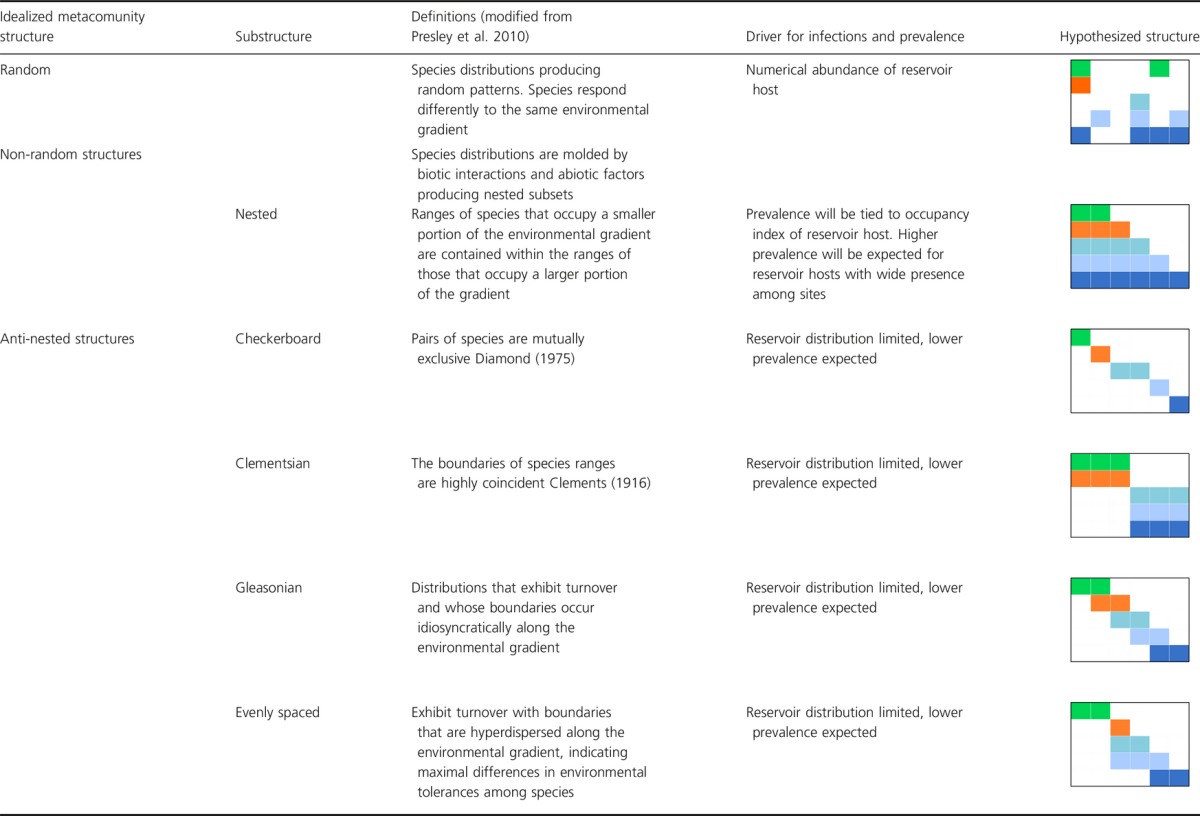
Definitions of metacommunity structures, substructures, and proposed mechanisms for infection and prevalence at each level, for each one of these structures and substructures. Colors represent different species, dark blue represents reservoirs

Each conceptual model of species distribution among sites was developed independently and was mostly descriptive. Clements (1916) conceived a structure in which unique communities had species with shared evolutionary history and ecological relationships. Thus, this results in species that share distinct communities along an environmental gradient. On the contrary, Gleason (1926) suggested a structure in which species had unique responses to environmental gradient coinciding with other species boundaries just by random. Diamond (1975) proposed strong competitive interactions would result in species pairs with nonoverlapping ranges thus resulting in a metacommunity checkerboard pattern when these pairs occur independently. Alternatively, in another scenario of heavy interspecific competition, tradeoffs among species would create even spacing in distributions along a gradient (Tilman 1982). Finally, nested subset of species in which communities is decreasing subsets from the most specious community will arise if species have differences in their inherent characteristics to disperse and colonize sites (Patterson and Attmar 1986). In the past, each one of these potential models was considered independent of each other, but newer analytical methods (Presley et al. 2010) can address concurrently the evaluation of metacommunity structure to distinguish among these possibilities. We propose this is crucial step for addressing disease ecology at the landscape level, as the distribution of host and nonhosts, create the metacommunity structure that will determine the distribution of the pathogen and its prevalence/incidence. We believe these recent analytical advances for the study of metacommunities are highly relevant as an initial step to understand pathogen dynamics at the regional level.

#### Human-dominated landscapes and environmental filtering

Changes in landscapes due to human activities have provided important insights into the dynamics of resulting communities. In general, these changes may favor competent reservoir host species, potentially boosting their distributions or abundances and thus increasing the risk of disease emergence. Little is known about how landscape changes relate to modification of metacommunity structure, subsequent reservoir distributions, and the occurrence of infectious diseases. We hypothesize that each metacommunity structure has different attributes that affect the spread and maintenance of a pathogen over the landscape. The possible outcomes are dependent on (1) the numerical abundance of the competent reservoir hosts and the pathogens; and (2) their occurrence in the metacommunity structure. On the one hand, in nested metacommunities the spatial coverage of sites (occupancy index) by the more competent host(s) is presumably the key factor determining pathogen prevalence in the landscape. On the other hand, antinested structures that exhibit a high variability in species composition among communities, which result in high species turnover, may be associated with lower pathogen prevalence over the landscape, as host and nonhost species' distributions tend to be limited to a subset of sites (Table2). Nevertheless, these theoretical expectations need empirical confirmation. This confirmation is not of mere academic interest as ascertaining these relationships could aid in disease management at the landscape level.

Anthropogenic changes in landscapes including habitat changes and fragmentation, and corresponding changes in climatic conditions can lead to local extinctions and colonization of host or nonhost species on the basis of their tolerance to the abiotic environment and adaptability to new conditions (environmental filtering, Table1). The species that are most tolerant to anthropogenic landscape changes are often the most competent, as well as the most abundant hosts (Mills 2006; Roche et al. 2012) (Fig.1). Moreover, these generalist host species are likely to occur throughout many of the local communities that form a metacommunity over a landscape (higher occupancy index, see Table2) with other more specialized host species using fewer number of sites, thus creating an overall nested pattern. Dispersion of competent host individuals would maintain pathogens at the landscape scale and less diverse communities at degraded sites would allow these generalist host species to increase in abundance, and thus, achieve higher prevalence of infection with pathogens that are present. Consequently, the risk of zoonotic diseases transmitted by generalist wildlife host species may be higher in disturbed areas. For example, in the Americas, habitat fragmentation and loss are homogenizing biodiversity in landscapes via local extinctions and have resulted in nested patterns of reservoir hosts for hantaviruses (Rubio et al. In press). The resulting landscape configuration associated with habitat loss and fragmentation has also been related to a change in infection dynamics and risk for multiple parasites (Suzán et al. 2012).

#### Phylogenetic diversity and biotic interactions at the landscape level

Animal communities are complex entities where a myriad of interactions among species is possible. For disease ecology, the most fruitful inquiries are often involve investigations of phylogenetically related host species, which are likely to evince strong ecological interactions, share pathogens, and provide opportunities for spillover, spillback, and host switching (Gaunt et al. 2001; Streicker et al. 2010; Medeiros et al. 2013). A novel approach that allows better understanding of the processes that produce patterns of biodiversity in communities and metacommunities is examination of the diversity of lineages of host and nonhost, (phylogenetic diversity; Cavender-Bares et al. 2009). Similar species are likely to exhibit similar traits and tolerance to ecological conditions (niche conservatism); hence, the distribution of species among communities may follow two general types of assembly rules derived from the possible interactions between phylogenetically related species.

A given metacommunity can exhibit phylogenetic overdispersion when the diversity of species represents a wider spectrum of lineages compared to random subsets of host species from the whole pool of species in the metacommunity (null models). Alternatively, a metacommunity may exhibit phylogenetic clustering when the lineages are less diverse than the null models (Webb et al. 2002). Utilizing these quantifiable patterns of phylogenetic diversity in conjunction with data on species richness and ecological traits, we can infer how communities were assembled (Leibold et al. 2010).

From the metacommunity perspective, processes such as active dispersal and environmental filtering constrain the community membership by selecting the host species that best survive and reproduce in the environment within each community. We predict that environmental filtering and niche-based processes (Table1) will generate phylogenetic clustering because closely related host species share similar niches (Fig.1). For example, while investigating communities of sunfishes in 890 lakes of Wisconsin, Helmus et al. (2007) found evidence that lakes with similar water quality contained closely related species that have similar tolerance to the environment. Likewise, we can suggest hypotheses about the prevalence of parasites across the landscape. Phylogenetic clustering of competent hosts due to changes in habitat suitability (e.g., environmental filtering and niche-based processes) may increase pathogen prevalence at the community level and increase variability of prevalence at the metacommunity level. Phylogenetic clustering of hosts and vectors suggests that some communities would be susceptible to a pathogen while other communities will include noncompetent hosts and thus be less susceptible. Theoretical and empirical evidence suggests that phylogenetic diversity of host(s) determines the niches available for a parasite. For example, mosquitoes of the genera *Culex* and *Aedes* are vectors of flaviviruses such as yellow fever, West Nile, Usutu, and dengue viruses. However, the biology and feeding preferences of these mosquito genera separate two large lineages (clades) of flaviviruses: the *Culex* clade transmitting viruses like West Nile, and the *Aedes* clade transmitting viruses like dengue (Gaunt et al. 2001). Hence, dengue viruses should only be effectively spread across communities in which *Aedes* mosquitoes are present.

While phylogenetic clustering may follow the patterns of diseases that are shared among phylogenetically related taxa (Davies and Pedersen 2008), phylogenetic overdispersion of parasites, vectors, and hosts suggests processes that are also of special interest in relation to the ecology of zoonotic diseases. When investigating parasite diversity in different fish species, it is possible to detect combinations of marine ectoparasites that will not infect the same species of fish (Poulin and Guégan 2000). Thus, assuming that niches are conserved through evolution, one might predict that antagonistic relationships would produce phylogenetic overdispersion because closely related host species are more likely to exclude each other than distantly related host species. However, no one has investigated phylogenetic overdispersion in parasite and pathogens and the evidence supporting the association between phylogenetic structure and antagonistic relationships between related species of hosts is scant. Although little is known about the relationships between phylogenetic diversity of hosts and infection of pathogens but research in this field is novel and promising (Streicker et al. 2010; Longdon et al. 2011; Medeiros et al. 2013). For example, by infecting 51 species of flies with sigma RNA-viruses (*Rhadboviridae*), Longdon et al. (2011) found that the effects of phylogenetic diversity in vector hosts were twofold. First, viruses were more likely to infect vector host species related to the original-natural hosts; and second, some lineages were more likely to be infected than others regardless of their relationship with the original host vector. This insightful evidence suggests that both phylogenetic diversity relative to the competent host vectors and phylogenetic diversity per se, influence the diversity of pathogens across the landscape. Hence, although it is plausible to argue that phylogenetic diversity influences disease dynamics and the prevalence of a pathogen, more studies are needed to disentangle the processes that phylogenetic diversity encompasses.

## Conclusions

Studies of the ecology of infectious diseases rarely address prevalence/incidence of pathogen infection in host species (i.e., vectors or reservoirs) at the landscape level (i.e., within and between communities across a region) by looking at the structural mechanisms of species assemblages. At the metacommunity scale, stochastic mechanisms and tolerance to ecological conditions of species that shape diversity between communities can result in both positive and negative correlations between host species diversity and prevalence of infectious diseases across a region (i.e., the metacommunity). Although the mechanisms driving diversity at the metacommunity scale are still unidentified, the distribution of host species across communities (i.e., metacommuntity structure) may facilitate or impede the distribution of their pathogens. Furthermore, to maintain human welfare and wildlife health it is fundamental to understand how anthropogenic changes, producing biodiversity loss and changes in ecosystem function can lead to the emergence of infectious diseases across communities. Using a metacommunity approach to investigate this question, we may elucidate emergent properties at the regional level that may drive risks of known infectious diseases and those yet to be discovered.

We have proposed a metacommunity-based conceptual framework and identified plausible mechanisms for shaping landscape-level patterns of diseases mechanisms that are commonly overlooked in disease ecology and epidemiology. We suggest that changes in dispersal, colonization, local extinctions, and biotic interactions resulting from changes in land use and ecosystem alteration are filtering and driving patterns of species distribution that subsequently can shape reservoir, vector, and infectious disease occurrences. These patterns can be mechanistically and spatially modeled with potentially large benefits for disease management. Because wildlife infectious disease dynamics, persistence, distribution, prevalence, and outbreaks all depend on ecological and phylogenetic diversity in a set of connected communities, the metacommunity represents an integrative framework for understanding disease ecology. Furthermore, as the portfolio of infectious disease spread and spatial distribution in metacommunities expands, public health professionals will be better able to evaluate the factors that predispose both a time and place to the origin and emergence of an infectious disease outbreak. This fundamental understanding will help improve the health of humans, wildlife, and domestic animals by mitigating the processes that drive the diversity of infectious disease threats we currently face and will continue to face into the future.

This conceptual model is a first step in establishing a metacommunity approach to infectious disease transmission. Mathematical models and empirical studies are needed to further understand the relevance and influence of metacommunities on infectious disease outbreaks and spread.
